# Pharmacological Activation of Rev-erb*α* Attenuates Doxorubicin-Induced Cardiotoxicity by PGC-1*α* Signaling Pathway

**DOI:** 10.1155/2023/2108584

**Published:** 2023-02-22

**Authors:** Runmei Zou, Shuo Wang, Hong Cai, Yuwen Wang, Cheng Wang

**Affiliations:** ^1^Department of Pediatric Cardiovasology, Children's Medical Center, The Second Xiangya Hospital, Central South University, Changsha, China; ^2^Department of Neonatology, Xiangya Hospital, Central South University, Changsha, China

## Abstract

**Background:**

Doxorubicin-induced cardiotoxicity has been closely concerned in clinical practice. Rev-erb*α* is a transcriptional repressor that emerges as a drug target for heart diseases recently. This study is aimed at investigating the role and mechanism of Rev-erb*α* in doxorubicin-induced cardiotoxicity.

**Methods:**

H9c2 cells were treated with 1.5 *μ*M doxorubicin, and C57BL/6 mice were treated with a 20 mg/kg cumulative dose of doxorubicin to construct doxorubicin-induced cardiotoxicity models in vitro and in vivo. Agonist SR9009 was used to activate Rev-erb*α*. PGC-1*α* expression level was downregulated by specific siRNA in H9c2 cells. Cell apoptosis, cardiomyocyte morphology, mitochondrial function, oxidative stress, and signaling pathways were measured.

**Results:**

SR9009 alleviated doxorubicin-induced cell apoptosis, morphological disorder, mitochondrial dysfunction, and oxidative stress in H9c2 cells and C57BL/6 mice. Meanwhile, PGC-1*α* and downstream signaling NRF1, TAFM, and UCP2 expression levels were preserved by SR9009 in doxorubicin-treated cardiomyocytes in vitro and in vivo. When downregulating PGC-1*α* expression level by specific siRNA, the protective role of SR9009 in doxorubicin-treated cardiomyocytes was attenuated with increased cell apoptosis, mitochondrial dysfunction, and oxidative stress.

**Conclusion:**

Pharmacological activation of Rev-erb*α* by SR9009 could attenuate doxorubicin-induced cardiotoxicity through preservation of mitochondrial function and alleviation of apoptosis and oxidative stress. The mechanism is associated with the activation of PGC-1*α* signaling pathways, suggesting that PGC-1*α* signaling is a mechanism for the protective effect of Rev-erb*α* against doxorubicin-induced cardiotoxicity.

## 1. Introduction

Doxorubicin, an anthracycline chemotherapy drug, is widely used to treat lymphoma, sarcoma, breast cancer, and pediatric leukemia [1]. Despite its efficacy in cancer chemotherapy, the cardiac complication induced by doxorubicin limits the use of this drug in clinical practice. A report suggested that a cumulative dose of 400, 550, or 550 mg/m^2^ led to heart failure incidence of 5%, 16%, and 26%, respectively [2]. Cardiomyocyte loss due to apoptosis and oxidative stress are the main causes of doxorubicin-induced cardiotoxicity [3, 4]. Mitochondrial dysfunction and reactive oxygen species (ROS) are thought to be the mechanism of doxorubicin-mediated cardiotoxicity [5]. Until now, earlier identification of risk factors, development of less-toxic derivatives, and earlier detection of subclinical toxicity may be helpful to prevent doxorubicin-induced cardiotoxicity [6]. However, there is no consensus on the best approach. It is urgent to find better cardioprotective strategies for prevention of doxorubicin-induced cardiotoxicity.

Rev-erb*α*, also known as NR1D1, is a core component of the circadian clock and functions as a transcriptional repressor. Rev-erb*α* recruits corepressors nuclear receptor corepressor 1(NCOR1) and histone deacetylase 3 (HDAC3) to inhibit the transcription of genes associated with circadian rhythm and metabolic diseases [7]. Recently, Rev-erb*α* emerges as a drug target for heart diseases [8]. Pharmacological activation of Rev-erb*α* prevents the development of cardiac hypertrophy and progression of advanced heart failure through transcription repression, indicating targeting Rev-erb*α* is a new approach to cardiac protection [9]. However, the role of Rev-erb*α* in doxorubicin-induced cardiotoxicity has not been investigated yet.

Peroxisome proliferator-activated receptor coactivator 1*α* (PGC-1*α*) is a transcriptional coactivator and is important in regulating mitochondrial energy homeostasis [10]. PGC-1*α* activation promotes the expression of downstream transcription factors, including nuclear respiratory factor 1 (NRF1), mitochondrial transcription factor A (TFAM), and uncoupling protein 2 (UCP2) [11, 12]. The previous study demonstrated that doxorubicin treatment suppressed the activation of PGC-1*α* pathway [13], suggesting PGC-1*α* signaling pathway is involved in doxorubicin-induced cardiotoxicity.

In this study, agonist SR9009 was used to activate Rev-erb*α*; then, the effect of activated Rev-erb*α* on doxorubicin-induced cardiotoxicity was investigated in vitro and in vivo. Further, PGC-1*α* signaling pathway was investigated to reveal the mechanism of activation of Rev-erb*α* on doxorubicin-induced cardiotoxicity.

## 2. Methods

### 2.1. Cell Culture, Treatment, and siRNA Transfection

Rat cardiomyocyte H9c2 cells were cultured in DMEM medium (HyClone, GE Healthcare) supplemented with 10% FBS (Gibco, Thermo Fisher Scientific, Inc., Waltham, USA), 100 U/mL penicillin-streptomycin, and 2 mM L-glutamine at 37°C in a humidified atmosphere with 5% CO_2_. Doxorubicin (MCE, New Jersey, USA) and SR9009 (APExBIO Technology, Houston, USA) stock solutions were preserved in DMSO and diluted in DMEM media before the experiment. H9c2 cells were plated on 6-well plates in antibiotic-free DMEM medium supplemented with FBS. Cells were transfected with scramble and PGC-1*α* siRNA (target sequence: TCATAAAGCCAACCAAGAT, Cat No. siB190925044252, RiboBio Co., Ltd., China) by Lipofectamine 2000 (Invitrogen, USA) when they reached 60-70% density. Cells were divided into six groups with three samples in each group. In the control group, cells were treated with an equal volume of DMSO; in the SR group, cells were treated with Rev-erb*α* agonist SR9009 (10 *μ*M) for 72 h; in the DOX group, cells were treated with 1.5 *μ*M doxorubicin for 72 h; in the DOX+SR group, cells were treated with SR9009 (10 *μ*M) and doxorubicin (1.5 *μ*M) for 72 h; in the DOX+SR+scramble siRNA group, cells were transfected with scramble siRNA for 48 h, treated with SR9009 (10 *μ*M) and doxorubicin (1.5 *μ*M) for 72 h; in the DOX+SR+PGC-1*α* siRNA group, cells were transfected with PGC-1*α* siRNA for 48 h, treated with SR9009 (10 *μ*M) and doxorubicin (1.5 *μ*M) for 72 h.

### 2.2. Animal Studies

Eight-week-old male C57BL/6 mice from the Animal Center of the Second Xiangya Hospital, Central South University, were utilized in the animal studies. The experiment was approved by the Animal Ethics Committee of the Second Xiangya Hospital (Approval No. 2020814). The mice were placed in a pathogen-free environment with the temperature of 20 ± 5°C and cycle of 12/12 hours light/dark and free to access food and water. Doxorubicin and SR9009 were preserved in DMSO and diluted by saline to final concentration of <2% DMSO (vehicle). C57BL/6 mice were randomly divided into four groups (*n* = 5 for each group). In the control group, the same volume of vehicle was intraperitoneally injected once a day from day 1 to day 8. In the SR group, SR9009 (100 mg/kg/d) was intraperitoneally injected once a day from day 1 to day 8. In the Dox group, doxorubicin (10 mg/kg/d) was intraperitoneally injected on day 2 and day 5 for a total of two times; at other times, the same volume of vehicle was intraperitoneally injected once a day. In the DOX+SR group, SR9009 was intraperitoneally injected at the dosage of 100 mg/kg/d every day from day 1 to day 8, and doxorubicin was intraperitoneally injected at the dosage 10 mg/kg/d on day 2 and day 5 for a total of two times. All C57BL/6 mice were euthanized on day 9, and heart tissues were collected for subsequent experiments.

### 2.3. Cell Viability Analysis

H9c2 cells were treated with different concentration of doxorubicin (0, 0.01, 0.1, 0.5, 1.0, 1.5, and 2 *μ*M) for 24, 48, and 72 h. Cell viability was determined by CCK-8 kit (Dojindo, Japan). Cells (5 × 10^3^ per well) were cultured in the 96-well plates in DMED medium at 37°C and treated with different concentrations of doxorubicin for 24, 48, and 72 h. Four parallel replicates were prepared. In each well, 10 *μ*L of CCK-8 was added to 100 *μ*L of medium and then incubated at 37°C for 4 h. Optical density (OD) values were detected at 450 nm by a microplate reader (BioTek, USA). Cell viability was expressed as the ratio of OD values of experimental wells to control wells.

### 2.4. Apoptosis Analysis

Apoptosis of H9c2 cells was analyzed by flow cytometry. Cells were resuspended in binding buffer, mixed with Annexin V-FITC and propidium iodide, and detected under a flow cytometer. Apoptosis of heart tissue was analyzed by TUNEL staining (Kaiji, China) according to the manufacturer's instructions. Cells were observed under a light microscope. The TUNEL-positive cells (brown) were counted in 5 randomly selected fields under high-power magnification. The apoptotic rate was expressed as the ratio between the number of TUNEL-positive cells and the total number of cardiomyocytes × 100.

### 2.5. Free Radical Production and ATP Content Analysis

Heart tissues were digested by mixed enzyme solution (0.4% type II collagenase: 0.125% trypsin = 2 : 1). Then, cell suspension was centrifuged and resuspended by DMEM medium supplemented with 10% FBS. DCF-DA staining was utilized to measure the generation of ROS. Cells were incubated with 10 *μ*M DCF-DA (Beyotime Bio., China) for 20 min (37°C). The fluorescent intensity was measured by a flow cytometer. Cells were lysed in the ATP assay buffer, homogenized, and centrifuged (4,000 rpm and 5 min) to pellet insoluble materials. ATP content was determined by mixing 300 *μ*L of the supernatant with 500 *μ*L of color-substrate solution. OD values were detected at 636 nm by a microplate reader.

### 2.6. Measurement of Malondialdehyde (MDA), Superoxide Dismutase (SOD), and Glutathione Peroxidase (GSH-Px)

The MDA content, SOD, and GSH-Px activities in cultured cells and heart tissues were measured using MDA, SOD, and GSH-Px assay kits according to the manufacturer's instructions. The OD values were analyzed by a microplate reader. Protein concentration determined by the bicinchoninic acid (BCA) assay (HonorGene, China) was utilized as internal standardization.

### 2.7. Immunofluorescent Staining and Hematoxylin-Eosin (HE) Staining

Cells were plated on the slide, washed three times with PBS, and fixed with 4% paraformaldehyde for 30 min. Travertine was added for penetration (37°C and 30 min). Cells were incubated with *α*-actin (Proteintech Group, Inc., USA; dilution fold 1 : 50) overnight (4°C) and washed three times with PBS. FITC-labeled goat anti-rabbit IgG (H + L) (Proteintech Group, Inc., USA; dilution fold 1 : 200) was added and incubated for 90 min (37°C). Nuclear was dyed with DAPI (37°C) for 10 min. Cell climbing film was viewed under a fluorescent microscope. After being taken off paraffin wax with xylene from paraffin-embedded heart tissue, heart tissue sections were hydrated with gradient ethanol, stained with hematoxylin-eosin, dehydrated with gradient ethanol and xylene, and mounted. The morphological changes of myocardial tissues were viewed under a light microscope.

### 2.8. Transmission Electron Microscopy

Mitochondrial ultrastructure of heart tissues was observed by transmission electron microscopy. Heart tissues of mice were collected, cut into 2 mm cubes, and fixed with 2.5% glutaraldehyde at 4°C for 24 h. After postfixation and dehydration, the samples were stained with uranium acetate. Subsequently, the samples underwent perfusion (acetone : Spurr = 3 : 1, 1 h, RT; acetone : Spurr = 1 : 1, 1-1.5 h, RT; and acetone : Spurr = 1 : 3, 1.5 h, RT), cut into ultrathin section of 70 nm thickness, and stained with lead citrate. Images were observed using transmission electron microscopy (Hitachi 7700).

### 2.9. Total RNA Extraction and RT-PCR

Total RNA was extracted using TRIzol reagent (Thermo, USA) and transcribed to cDNA by mRNA reverse transcription kit (Kangwei, China). RT-PCR was performed by UltraSYBR Mixture (Kangwei, China). The primer sequences involved in this study are listed in Table 1. The 2−*ΔΔ*Ct method was utilized to analyze the relative fold change of the transcripts, and the endogenous mRNA level of GAPDH or actin was applied for internal standardization.

### 2.10. Western Blot

Cells and heart tissues were prepared, and the proteins were extracted according to the manufacturers' protocol. Protein concentration was determined by BCA protein assay (HonorGene, China). SDS-PAGE, transblotting, and subsequent immunodetection were performed as previously described [14]. Blots were incubated with primary antibodies against PGC1*α* (AB3242, 1 : 1000, MilliporeSigma), TFAM (22586-1-AP, 1 : 2000, Proteintech, USA), NRF1 (12482-1AP, 1 : 1000, Proteintech, USA), UCP2 (11081-1-AP, 1 : 1000, Proteintech, USA), Bcl2 (26593-1-AP, 1 : 2000, Proteintech, USA), caspase 3 (19677-1-AP, 1 : 1000, Proteintech, USA), and GAPDH (10494-1-AP, 1 : 3000, Proteintech, USA) that were incubated overnight at 4°C. Then, the membranes were washed by PBST three times and incubated with HRP goat anti-rabbit IgG (SA00001-2, 1 : 6000, Proteintech, USA) at room temperature for 1.5 h. GAPDH was used as an internal control. Levels of target protein within bands were determined using Quantity One software.

## 3. Results

### 3.1. SR9009 Alleviated Doxorubicin-Induced Cardiomyocyte Hypertrophy, Mitochondrial Oxidative Stress, and Apoptosis of H9c2 Cells

**Figure 1:**
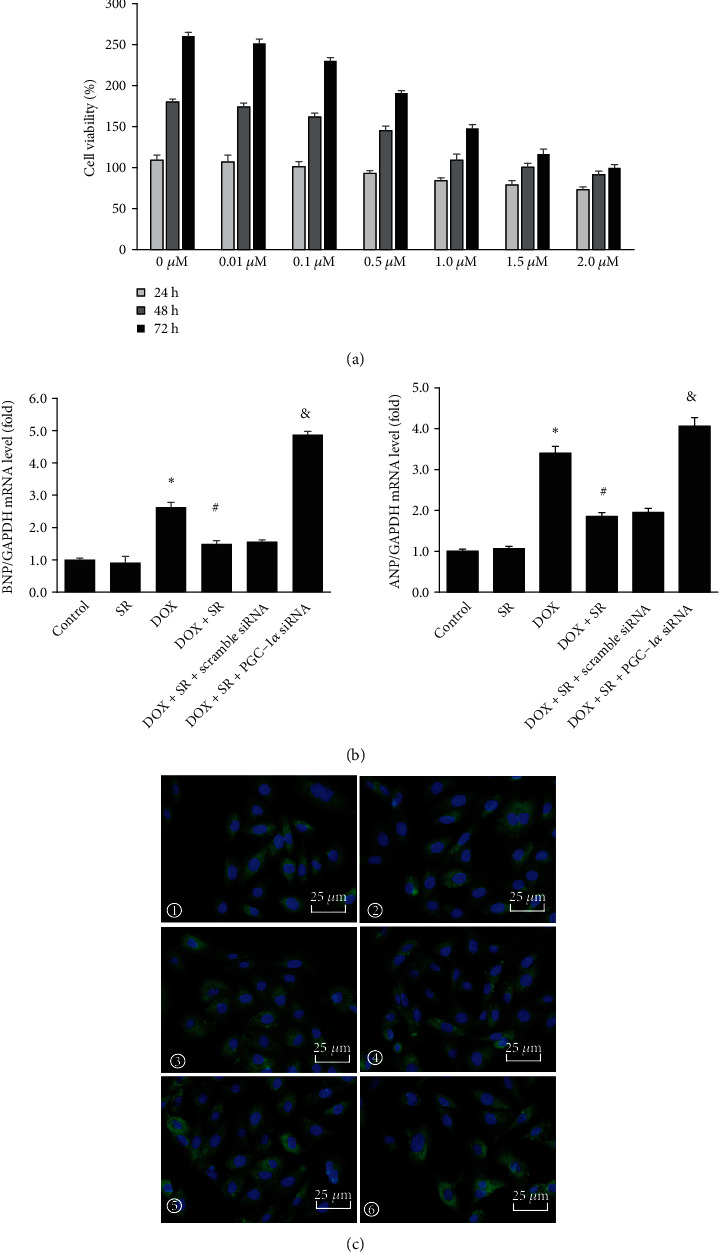
Effects of SR9009 and PGC-1*α* siRNA on cell viability and cardiomyocyte hypertrophy in doxorubicin-treated H9c2 cells. (a) Cell viability with different concentration of doxorubicin for 24, 48, and 72 h. (b) ANP and BNP mRNA expression level in H9c2 cells. (c) Immunofluorescent staining with *α*-actin (green), nuclear is dyed with DAPI (blue); scale bar: 25 *μ*m. ① Control; ② SR9009; ③ DOX; ④ DOX+SR; ⑤ DOX+SR+scramble siRNA; ⑥ DOX+SR+PGC-1*α* siRNA. The results are expressed as mean ± SD, *n* = 3. ^∗^Compared with the control group, *P* < 0.05. ^#^Compared with the DOX group, *P* < 0.05. ^&^Compared with the DOX+SR+scramble siRNA group, *P* < 0.05.

CCK-8 assay was applied to detect doxorubicin-treated cell viability. H9c2 cell viability was inhibited by doxorubicin in a dose-dependent manner (Figure 1(a), *P* < 0.05). Cell viability was reduced to 55.3 ± 2.7% by treatment of 1.5 *μ*M doxorubicin for 72 h. 1.5 *μ*M doxorubicin was used in the following experiments. Doxorubicin treatment for 72 h increased cardiomyocyte hypertrophy marker atrial natriuretic peptide (ANP) and brain natriuretic peptide (BNP) mRNA levels (vs. control group, *P* < 0.05) whereas SR9009 treatment could decrease doxorubicin-induced ANP and BNP mRNA levels (vs. DOX group, *P* < 0.05, Figure 1(b)). Immunofluorescent staining suggested that doxorubicin-treated H9c2 cells were enlarged and reversed by treatment of SR9009 (Figure 1(c)).

**Figure 2:**
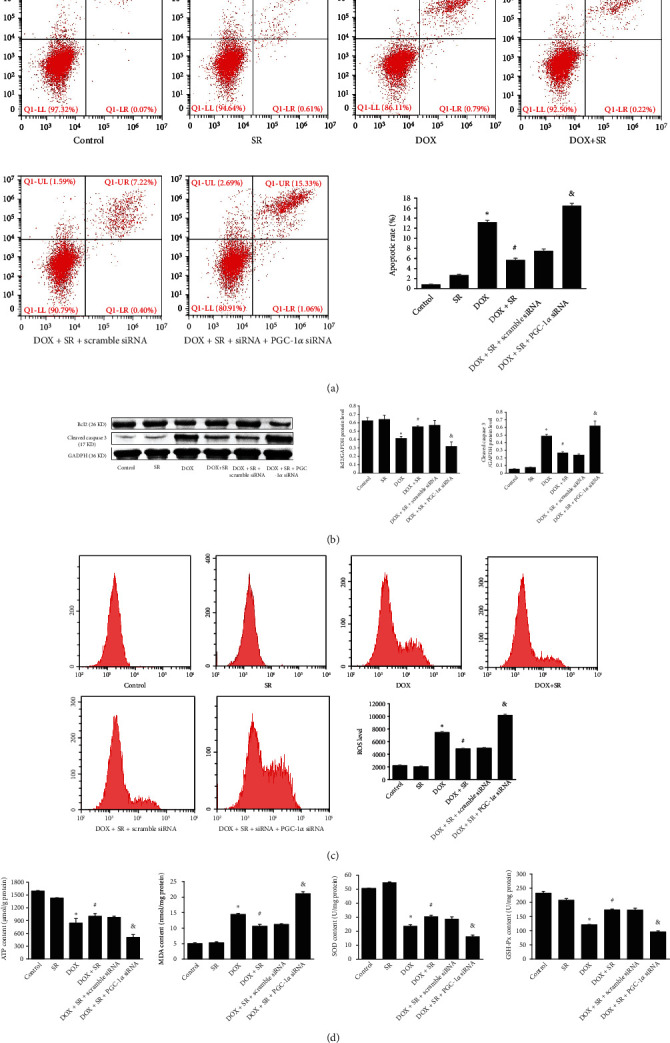
Effects of SR9009 and PGC-1*α* siRNA on cell apoptosis, ROS generation, cellular ATP content, MDA content, and SOD and GSH-Px activities in doxorubicin-treated H9c2 cells. (a) Representative images of cell apoptosis analyzed by flow cytometry. The horizontal axis represents Annexin V-FITC, and the vertical axis represents propidium iodide. (b) Representative western blot results of Bcl2 and cleaved caspase 3 are shown; GAPDH was used as an internal control. (c) Representative images of ROS generation analyzed by flow cytometry. The horizontal axis represents fluorescent intensity, and the vertical axis represents cell count. (d) Cellular ATP content, MDA content, SOD activity, and GSH-Px activity. The results are expressed as mean ± SD, *n* = 3. ^∗^Compared with the control group, *P* < 0.05. ^#^Compared with the DOX group, *P* < 0.05. ^&^Compared with the DOX+SR+scramble siRNA group, *P* < 0.05.

Cell apoptosis was analyzed by flow cytometry. The apoptotic rate of H9c2 cells was significantly increased by doxorubicin treatment when compared with control group (*P* < 0.05, Figure 2(a)). However, doxorubicin-induced apoptotic rate was alleviated when cotreatment of doxorubicin and SR9009 (vs. DOX group, *P* < 0.05, Figure 2(a)). Consistent with these results, doxorubicin decreased Bcl2 protein levels and increased cleaved caspase 3 protein level (vs. control group, *P* < 0.05, Figure 2(b)), while cotreatment of doxorubicin and SR9009 reserved antiapoptotic Bcl2 protein levels and reduced cleaved caspase 3 protein level when compared with the DOX group (*P* < 0.05, Figure 2(b)).

Intracellular ROS production was analyzed by DCFH method. Doxorubicin treatment increased the generation of ROS and decreased cellular ATP content (vs. control group, both *P* < 0.05, Figures 2(c) and 2(d)), whereas cotreatment of SR9009 decreased doxorubicin-induced ROS production and reserved cellular ATP content (vs. DOX group, both *P* < 0.05, Figures 2(c) and 2(d)). GSH-Px and SOD are endogenous defenses against oxidative injury, and MDA is an oxidative stress indicator. Doxorubicin exposure for 72 h decreased GSH-Px and SOD activities and increased MDA content when compared with the control group (all *P* < 0.05, Figure 2(d)). However, SR9009 preserved doxorubicin-induced decrease of GSH-Px and SOD activities and reduced MDA content (vs. DOX group, all *P* < 0.05, Figure 2(d)). These results suggested that SR9009 alleviates doxorubicin-induced mitochondrial oxidative stress.

### 3.2. SR9009 Activated PGC-1*α* and Downstream Signaling in DOX-Treated H9c2 Cells

**Figure 3:**
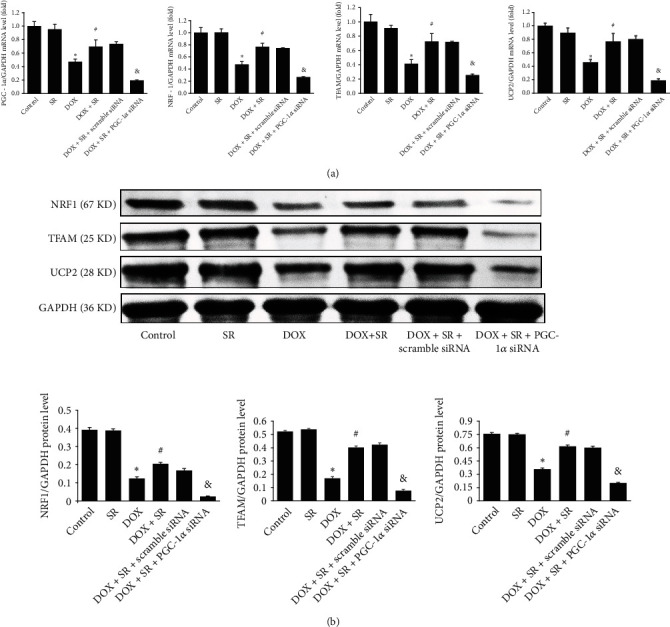
Effects of SR9009 and PGC-1*α* siRNA on PGC-1*α* signaling in doxorubicin-treated H9c2 cells. (a) mRNA levels of PGC-1*α*, NRF1, TFAM, and UCP2 measured by RT-PCR. (b) Representative western blot result of NRF1, TFAM, UCP2, and GAPDH was used as an internal control. The results are expressed as mean ± SD, *n* = 3. ^∗^Compared with the control group, *P* < 0.05. ^#^Compared with the DOX group, *P* < 0.05. ^&^Compared with the DOX+SR+scramble siRNA group, *P* < 0.05.

To further investigate the role of SR9009 in cardiac protection, PGC-1*α* and downstream signaling NRF1, TFAM, and UCP2 expression levels were measured. PGC-1*α*, NRF1, TFAM, and UCP2 mRNA levels were obviously reduced by doxorubicin (vs. control group, all *P* < 0.05, Figure 3(a)) and remarkably augmented by cotreatment of doxorubicin and SR9009 (vs. DOX group, all *P* < 0.05, Figure 3(a)). Consistent with these results, NRF1, TFAM1, and UCP2 protein levels were also reduced by doxorubicin but reserved by SR9009 in doxorubicin-treated cells (all *P* < 0.05, Figure 3(b)).

### 3.3. Effects of SR9009 on Cardiac Morphology, Mitochondrial Oxidative Stress, Apoptosis, and PGC-1*α* and Downstream Signaling in Doxorubicin-Treated Mouse Heart

**Figure 4:**
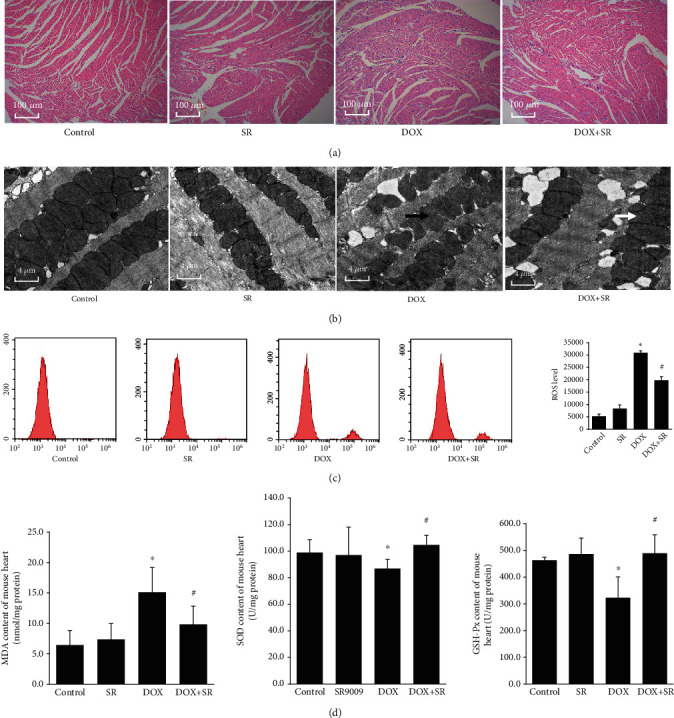
Effects of SR9009 on cardiotoxicity in doxorubicin-treated mouse heart. (a) Representative HE staining images of left ventricle. Scale bar: 100 *μ*m. (b) Representative ultrastructural morphology images of mitochondria of left ventricle. Scale bar: 4 *μ*m. Black arrows: swollen mitochondria with cristae disorientation and breakage; white arrowheads: normal mitochondria. (c) Representative ROS generation of heart tissue analyzed by flow cytometry. The horizontal axis represents fluorescent intensity, and the vertical axis represents cell count. (d) MDA content, SOD activity, and GSH-Px activity of heart tissue. The results are expressed as mean ± SD, *n* = 5. ^∗^Compared with the control group, *P* < 0.05. ^#^Compared with the DOX group, *P* < 0.05.

To further reveal the protective role of SR9009 in doxorubicin-induced cardiotoxicity in vivo, doxorubicin-induced mouse model was used. HE staining demonstrated that treatment of doxorubicin on C57BL/6 mice induced degeneration of myocardial tissues, whereas cotreatment of doxorubicin and SR9009 alleviated this effect (Figure 4(a)).

Transmission electron microscopy examination indicated that doxorubicin exposure induced significant disorders of ultrastructural mitochondrial morphology. In doxorubicin-treated mouse heart tissue, mitochondrial swelling with cristae disorientation and breakage was observed. SR9009 cotreatment significantly alleviated mitochondrial morphological disorder (Figure 4(b)). Consistent with the above results, doxorubicin treatment increased ROS production of mouse heart tissue (vs. control group, *P* < 0.05), whereas cotreatment of SR9009 obviously decreased ROS generation compared with the DOX group (*P* < 0.05, Figure 4(c)). Cotreatment of SR9009 and doxorubicin decreased doxorubicin-induced increase in MDA content and increased doxorubicin-induced decrease in SOD and GSH-Px activities in mouse heart tissue (all *P* < 0.05, Figure 4(d)).

**Figure 5:**
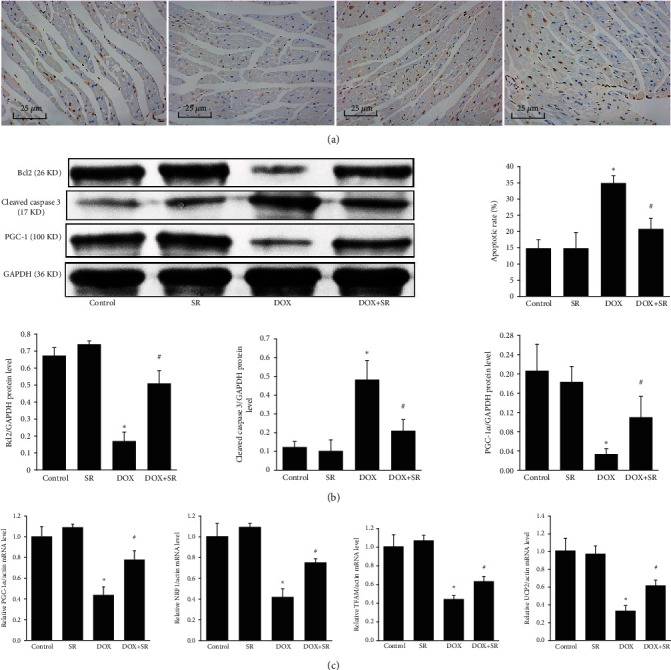
Effects of SR9009 on cell apoptosis and PGC-1*α* signaling pathway in doxorubicin-treated mouse heart. (a) Representative image of TUNEL staining. The apoptotic rate was expressed as the ratio between the number of TUNEL-positive cells (brown) and the total number of cardiomyocytes × 100%. Scale bar: 25 *μ*m. (b) Representative western blot result of Bcl2, cleaved caspase 3, PGC-1*α*, and GAPDH was used as an internal control. (c) mRNA levels of PGC-1*α*, NRF1, TFAM, and UCP2 measured by RT-PCR. The results are expressed as mean ± SD, *n* = 5. ^∗^Compared with the control group, *P* < 0.05. ^#^Compared with the DOX group, *P* < 0.05.

TUNEL assay indicated that doxorubicin exposure raised apoptotic rate compared with the control group, while cotreatment of SR9009 alleviated doxorubicin-induced apoptosis in mouse heart tissue (*P* < 0.05, Figure 5(a)). We measured Bcl2 and cleaved caspase 3 expression level. The results demonstrated that cotreatment of doxorubicin and SR9009 increased Bcl2 protein levels and decreased cleaved caspase 3 protein levels (vs. DOX group, all *P* < 0.05, Figure 5(b)).

Further, we detected the expression of PGC-1*α* and downstream signaling in mouse heart. PGC-1*α* and downstream signaling NRF1, TFAM, and UCP2 expression levels were significantly decreased in doxorubicin-treated mouse heart and increased when coexposure of doxorubicin and SR9009 (all *P* < 0.05, Figures 5(b) and 5(c)). These results suggested that Rev-erb*α* might protect heart against doxorubicin-induced cardiotoxicity by regulating PGC-1*α* signaling.

### 3.4. Downregulation of PGC-1*α* on the Effects of Cardiomyocyte Hypertrophy, Mitochondrial Oxidative Stress, Apoptosis, and PGC-1*α* Signaling in Doxorubicin- and SR9009-Co-Treated H9c2 Cells

To investigate the role of PGC-*α* signaling in cardioprotection of Rev-erb*α* against doxorubicin-induced cardiotoxicity, PGC-1*α* expression level was downregulated by specific siRNA (Figure S1). In doxorubicin- and SR9009-co-treated H9c2 cells, downregulation of PGC-1*α* by siRNA increased ANP and BNP mRNA levels and enlarged the size of H9c2 cells as shown by immunofluorescence staining (Figures 1(b) and 1(c)). As shown in Figure 2, compared with scramble siRNA, apoptotic rate of H9c2 cells and cleaved caspase 3 protein level were raised when downregulating PGC-1*α* in doxorubicin- and SR9009-co-treated cells. On the contrary, antiapoptosis factor Bcl2 protein level was remarkably alleviated by knockdown of PGC-1*α*. In doxorubicin- and SR9009-co-treated H9c2 cells, ROS generation was increased and cellular ATP content was decreased by PGC-1*α* siRNA compared with scramble siRNA (*P* < 0.05). Unsurprisingly, when downregulating PGC-1*α*, MDA content was increased; GSH-Px and SOD contents were obviously decreased in doxorubicin- and SR9009-co-treated cells (vs. scramble siRNA, all *P* < 0.05, Figure 2(d)). PGC-1*α* downstream signaling NRF1, TFAM, and UCP2 mRNA and protein levels were significantly reduced by PGC-1*α* siRNA in doxorubicin- and SR9009-co-treated H9c2 cells (vs. scramble siRNA, *P* < 0.05, Figure 3).

## 4. Discussion

Doxorubicin has been used to treat pediatric and adult cancer for decades. However, the cardiotoxicity of doxorubicin hampered its clinical use. Several mechanisms associated with doxorubicin-induced cardiotoxicity, among which apoptosis mediated loss of cardiomyocytes and cardiac oxidative stress, are the most important. The use of cardioprotective agent is one of the approaches for primary prevention of doxorubicin-induced cardiotoxicity. In the present study, we found that pharmacological activation of Rev-erb*α* by SR9009 decreased doxorubicin-induced cell apoptosis and oxidative stress in vivo and vitro, suggesting that Rev-erb*α* has a protective role against doxorubicin-induced cardiotoxicity.

ROS plays a crucial role in doxorubicin-induced cardiotoxicity. Imbalance between the generation of ROS, reactive nitrogen species (RNS), and intrinsic antioxidant mechanism results in accumulation of ROS and RNS in cardiomyocytes and is not effectively removed by the inherent antioxidant mechanism [15]; then, oxidative stress increased. Mitochondrial dysfunction is believed to be a pathological mechanism for doxorubicin-induced cardiotoxicity and plays a key role in the progression of cardiotoxicity [16]. Mitochondria accounts for 90% ATP production in cardiomyocytes. During the treatment of doxorubicin, ROS production is increased by reduction of the redox cycling in complex I of electron transport chain, which results in disruption of ultrastructure of mitochondria and irreversible myocardial oxidative injury [17, 18].

Rev-erb*α*, belonging to a nuclear receptor superfamily, regulates circadian rhythm, glucose and lipid metabolism, and inflammatory response. Rev-erb*α* agonist SR9009 improved skeletal muscle oxidative capacity through regulating mitochondrial number and autophagy [19]. Rev-erb*α* increased mitochondrial biogenesis, reduced mitophagy, and protect cells against oxidative stress [20]. In the present study, Rev-erb*α* was pharmacologically activated by SR9009. Doxorubicin-induced disorders of ultrastructural mitochondrial morphology were reversed, and ATP production was increased by SR9009. Further, treatment of SR9009 decreased doxorubicin-induced cell apoptosis and oxidative stress level both in mouse heart tissue and H9c2 cells. These results suggested that Rev-erb*α* has a protective role against doxorubicin-induced oxidative stress and cell apoptosis.

**Figure 6:**
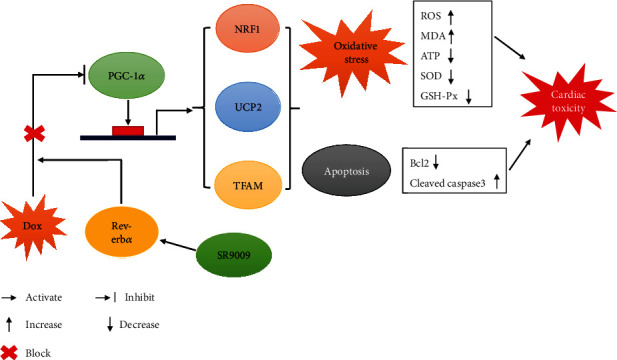
A schematic representation of SR9009-activated Rev-erb*α* which protects against doxorubicin-induced cardiotoxicity through PGC-1*α* signaling. Activation of Rev-erb*α* by SR9009 preserves PGC-1*α* and downstream signaling NRF1, UCP2, and TFAM, which further alleviates oxidative stress and apoptosis, consequently contributing to inhibition of DOX-induced cardiotoxicity.

PGC-1*α* is important in regulating mitochondrial energy homeostasis by inducing the expression of NRF1, TFAM, and UCP2. NRF1 plays a pivotal role in maintaining mitochondrial function by transcriptional regulation of genes associated with respiratory complexes and mitochondrial enzymes [21]. TFAM is one of the mitochondrial transcription factors and regulates mtDNA transcription and replication [22]. UCP2 is a mitochondrial inner membrane protein that can alleviate generation of mitochondrial ROS [23]. Studies demonstrated that doxorubicin caused inhibition of PGC-1*α* expression/activity in cardiomyocytes and accelerated doxorubicin-induced cardiotoxicity [24, 25]. PGC-1*α* stimulates Rev-erb*α* expression through coactivation of the ROR family of orphan nuclear receptor to regulate circadian rhythm and energy metabolism [26]. Whether the protective effect of Rev-erb*α* on doxorubicin-induced cardiotoxicity is mediated through PGC-1*α* signaling was revealed in our study. PGC-1*α* and downstream transcription factors NRF1, TFAM, and UCP2 expression levels were decreased by treatment of doxorubicin and preserved when cotreatment of doxorubicin and SR9009 in vivo and vitro. Further, PGC-1*α* expression level was downregulated by siRNA in H9c2 cells; cell apoptosis and oxidative stress levels were increased in SR9009- and doxorubicin-treated cells compared with scramble siRNA. Not surprisingly, NRF1, TFAM, and UCP2 expression levels were decreased. These results suggested that activation of Rev-erb*α* by SR9009 might alleviate doxorubicin-induced cardiotoxicity through coactivator PGC-1*α* (Figure 6).

PGC-1*α* gene expression is regulated by adenosine monophosphate-activated protein kinase (AMPK), especially under physiological stress conditions [27]. AMPK/PGC-1*α* signaling is supposed to be a potential myocardial protective target against doxorubicin-induced cardiotoxicity [28]. In this study, we found that pharmacological activation of Rev-erb*α* activated PGC-1*α* and downstream signaling NRF1, TFAM, and UCP2 to preserve mitochondrial function and alleviate oxidative stress in doxorubicin-treated cardiomyocytes. However, whether AMPK is activated should be investigated in future study. Besides, overexpression or deficiency transgenic mice should be utilized to reveal the role of Rev-erb*α* in doxorubicin-induced cardiotoxicity.

In conclusion, pharmacological activation of Rev-erb*α* by SR9009 could attenuate doxorubicin-induced cardiotoxicity through preservation of mitochondrial function and alleviation of apoptosis and oxidative stress. The mechanism is associated with activation of PGC-1*α* signaling pathways, suggesting that PGC-1*α* signaling is a mechanism for the protective effect of Rev-erb*α* against doxorubicin-induced cardiotoxicity.

## Figures and Tables

**Table 1:** Primers used in the study.

Genes	Sequences (5′ to 3′)
rat-GAPDH	F-ACAGCAACAGGGTGGTGGAC
	R-TTTGAGGGTGCAGCGAACTT
rat-PGC-1*α*	F-AATCAAGCCACTACAGACACC
	R-TCTCTGCGGTATTCGTCCCTC
rat-NRF1	F-TTGAGTCTAACCCATCTATCCG
	R-TGTCCCACTCGTGTCGTAT
rat-TFAM	F-AGAAACGCCTAAAGAAGAA
	R-TCCAAGCCTGATTTACAAG
rat-UCP2	F-CAATGTTGCCCGAAATGC
	R-CAAGGGAGGTCGTCTGTC
rat-Bcl2	F-CTGGTGGACAACATCGCTCT
	R-ATAGTTCCACAAAGGCATCCCA
rat-ANP	F-GGATTTCAAGAACCTGCTAGACCAC
	R-CTTCATCGGTCTGCTCGCTCA
rat-BNP	F-AGATGGCACATAGTTCAAGC
	R-AAAACAACCTCAGCCCGTCA
mouse-actin	F-ACATCCGTAAAGACCTCTATGCC
	R-TACTCCTGCTTGCTGATCCAC
mouse-PGC-1*α*	F-AATCAAGCCACTACAGACACC
	R-TTTCAGACTCCCGCTTCTCG
mouse-NRF1	F-CGCAGCACCTTTGGAGAA
	R-CCCGACCTGTGGAATACTTG
mouse-TFAM	F-CAAAGGATGATTCGGCTCAGG
	R-TCGACGGATGAGATCACTTCG
mouse-UCP2	F-AAAGCAGCCTCCAGAACTCC
	R-ATTCTGATTTCCTGCTACCTCC
mouse-Bcl2	F-TTGAAAACCGAACCAGGAATTGC
	R-GTCCTGTGCCACTTGCTCT

## Data Availability

The data used to support the findings of this study are available from the corresponding author upon request.

## Supplementary Materials

Supplementary Materials Figure S1: PGC-1*α* mRNA and protein expression level. H9c2 cells were transfected with PGC-1*α* siRNA001/002/003; mRNA (A) and protein (B) level was measured by RT-PCR and western blot. PGC-1*α* expression level was significantly decreased by siRNA001. So PGC-1*α* siRNA001 was used in the experiment. Results are expressed as mean ± SD, *n* = 3. ^∗^Compared with scramble siRNA, *P* < 0.05.
