# Supplementary material for: Pharmacological Activation of Rev-erbα Attenuates Doxorubicin-Induced Cardiotoxicity by PGC-1α Signaling Pathway
Source: Cardiovasc Ther. 2023 Feb 22;2023:2108584. doi: 10.1155/2023/2108584 (PMC9977526; doi:10.1155/2023/2108584)
Supplement: Supplementary Materials — Figure S1: PGC-1α mRNA and protein expression level. H9c2 cells were transfected with PGC-1α siRNA001/002/003; mRNA (A) and protein (B) level was measured by RT-PCR and western blot. PGC-1α expression level was significantly decreased by siRNA001. So PGC-1α siRNA001 was used in the experiment. Results are expressed as mean ± SD, n = 3. ∗Compared with scramble siRNA, P < 0.05. [file 2108584.f1.zip › Figure S1.pptx]

## Slide 1
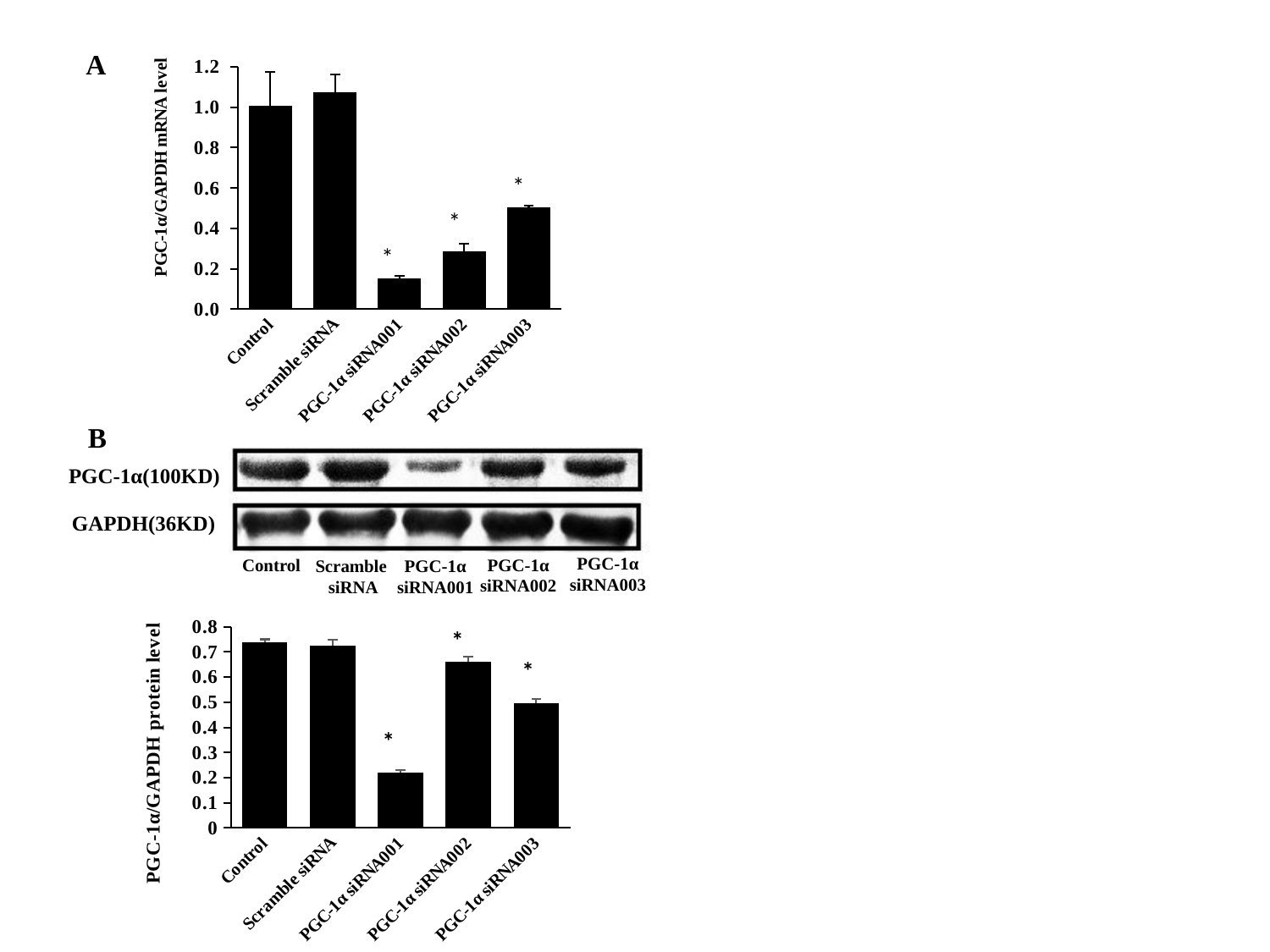

A
### Chart
| Category | |
|---|---|
| Control | 1.0086918284912167 |
| Scramble siRNA | 1.0718016394897207 |
| PGC-1α siRNA001 | 0.1504444248108209 |
| PGC-1α siRNA002 | 0.28665183767436714 |
| PGC-1α siRNA003 | 0.5058610834407332 |*
B
PGC-1α(100KD)
GAPDH(36KD)
PGC-1α
siRNA003
Control
PGC-1α
siRNA002
Scramble
siRNA
PGC-1α
siRNA001
### Chart
| Category | |
|---|---|
| Control | 0.7379360348022236 |
| Scramble siRNA | 0.7252657815758686 |
| PGC-1α siRNA001 | 0.21982320269107405 |
| PGC-1α siRNA002 | 0.6596493801804444 |
| PGC-1α siRNA003 | 0.497050349842228 |*
*
