# Supplementary material for: Pharmacological Activation of Rev-erbα Attenuates Doxorubicin-Induced Cardiotoxicity by PGC-1α Signaling Pathway
Source: Cardiovasc Ther. 2023 Feb 22;2023:2108584. doi: 10.1155/2023/2108584 (PMC9977526; doi:10.1155/2023/2108584)
Supplement: Supplementary Materials — Figure S1: PGC-1α mRNA and protein expression level. H9c2 cells were transfected with PGC-1α siRNA001/002/003; mRNA (A) and protein (B) level was measured by RT-PCR and western blot. PGC-1α expression level was significantly decreased by siRNA001. So PGC-1α siRNA001 was used in the experiment. Results are expressed as mean ± SD, n = 3. ∗Compared with scramble siRNA, P < 0.05. [file 2108584.f1.zip › supplementary file (1).docx]

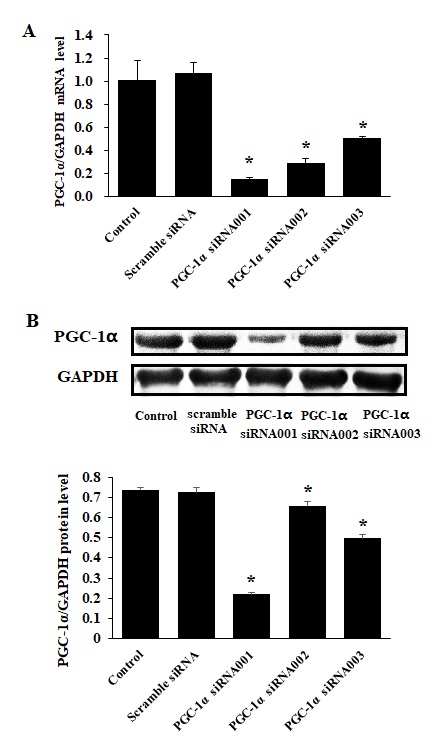


Figure S1 PGC-1α mRNA and protein expression level. H9c2 cells were transfected with PGC-1α siRNA 001,002,003, mRNA (A) and protein (B) level was measured by RT-PCR and western blot. PGC-1α expression level was significantly decreased by siRNA001. So PGC-1α siRNA001 was used in the experiment. The results are expressed as the mean± SD, n=3. * compared with scramble siRNA, *P*＜0.05.
